# Genetic parameters and selection strategies for soybean genotypes resistant to the stink bug-complex

**DOI:** 10.1590/S1415-47572009000200020

**Published:** 2009-06-01

**Authors:** Cláudio Roberto Cardoso de Godoi, José Baldin Pinheiro

**Affiliations:** 1 Nidera Sementes Ltda., Soybean Program Research, Rio Verde, GoiásBrazil; 2 Departamento de Genética, Escola Superior de Agricultura Luiz de Queiroz, Universidade de São Paulo, Piracicaba, SPBrazil

**Keywords:** *Glycine max*, insect resistance, stink bugs, heritability

## Abstract

Soybean genotypes resistant to stink bugs are derived from complex breeding processes obtained through indirect selection. The aim of the present work was to estimate genetic parameters for guiding selection strategies towards resistant genotypes, based on those traits associated with responses to pod-attacking stink bugs, such as the grain filling period (GFP), leaf retention (LR), percentage index of pod damage (PIPD) and percentage of spotted seeds (PSS). We assessed the parental lines IAC-100 (resistant) and FT-Estrela (susceptible), the progenies F_2_ and F _4_ , 30 progenies F _2:3_ , 30 progenies BC _1_ F _2:3_ and 30 progenies BC _2_ F _2:3_ , besides the cultivars BRS Celeste and MGBR-46 (Conquista). Three field experiments, using randomized complete block design with three replications, were installed in Goiânia-GO, in the 2002/03 season. Each experiment consisted of 36 treatments (6 common and 30 regular). Heritability estimates were: 74.6 and 36.1 (GFP); 51.9 and 19.9 (LR); 49.6 and 49.6 (PIPD) and 55.8 and 20.3 (PSS), in both the broad and narrow senses, respectively. Based on these results, we concluded that the best strategy for obtaining stink bug-resistant genotypes consists of selecting the PIPD trait in early generations (F _3_ or F _4_ ), followed by selection for the GFP, LR and PSS traits in generations with higher endogamy levels.

## Introduction

Stink bugs are considered to be the most important pests attacking soybean. The group of species that most frequently causes economic losses is called the “stink bug complex”, composed of three species: *Nezara viridula, Piezodorus guildinii* and *Euchistus heros.* Through their piercing-sucking feeding habits, these insects cause damage mainly during pod formation, filling and maturation (Gazzoni, 1998; Lourenção *et al.* 2002).

The selection of genotypes resistant to chewing and sucking-insects has been basically carried out among lines in populations consisting of F_3_ to F_5_ generations. Genotype evaluation is done by scoring the plants at the reproductive stage, according to defoliation scales for chewing insects, and/or leaf retention scores associated to the presence of empty pods on the upper third of the plants at the maturation stage for sucking insects, such as the stink bugs (Lourenção *et al.*, 2002). Resistant genotypes have also been obtained through indirect selection of those with shorter grain-filling periods, lower percentage of spotted seeds, lower hundred - seeds weight and lower indexes of pod damage (Godoi *et al.*, 2002; Moura and Pinheiro, 2002; Pinheiro *et al.*, 2005).

Indirect selection has also been widely used to obtain insect-resistant soybean genotypes, with satisfying results as to selection gains, pre-breeding lines and cultivars, notably the cultivar IAC-100, an example of resistance to several insect species preying on soybean crops (Souza and Toledo, 1995; Rossetto *et al.*, 1995; Pinheiro *et al.*, 2005). In general, the phenotypic traits employed in indirect selection of those genotypes resistant to defoliation and sucking insects in soybeans have been reduced defoliation levels, a shorter pod-filling period, lower indices of pod damage, lower weight of a hundred seeds and lower levels of seed damage (Miranda *et al.*, 1979; Rossetto *et al.*, 1986; Souza and Toledo, 1995; Godoi *et al.*, 2002; Moura and Pinheiro, 2002; Moura *et al.*, 2003; Pinheiro *et al.*, 2005). According to Rossetto *et al.* (1995), these traits represent resistance mechanisms against stink bugs in soybeans. The authors list certain mechanisms resorted to in soybeans against stink bug attacks, as for example, a shorter pod-filling period, a higher seed-yield per plant, the capacity to reject damaged immature pods and their substitution by new pods, besides normal senescence with leaf dropping at the maturation stage and resistance to the yeast *Nematospora coryli* transmitted by stink bugs.

The study of those genetic mechanisms associated to stink bug resistance in soybeans reveals that, although the traits involved are subject to complex genetic control, it is possible to obtain superior descendants from crosses between susceptible and resistant genotypes. Thus, soybean breeding aiming at reducing stink bug vulnerability is a promising field for research, since selection in segregated populations has permitted obtaining gains in this area (Souza and Toledo, 1995). However, it is noteworthy that traits related to insect resistance are quantitative and should be allied to yield, another quantitative trait, though of low heritability, thereby making it difficult to obtain resistant and, at the same time, high-yield genotypes.

Although resistant lines and cultivars can be successfully obtained, only few studies have been carried out with the purpose of revealing the genetic mechanisms underlying these traits and the genetic parameters associated thereto. According to Vencovsky and Barriga (1992), genetic studies in breeding programs are important, through disclosing the genetic basis and inheritance of a given trait under selection, thereby giving rise to the choice of the most adequate methods for managing segregating populations in order to obtain better results and for determining the most adequate stage for undertaking selection, with a view to higher gains. The availability of this information can significantly contribute to improving breeding programs. Thus, the purpose manifest in the present work was to estimate genetic parameters associated to resistance against sucking stink bugs in soybean pods, in order to investigate genetic control and indicate selection strategies for obtaining resistant genotypes based on these traits.

## Material and Methods

The parent lines FT-Estrela and IAC-100 constituted the plant material used in this study. The cultivar FT-Estrela, used as the stink bug susceptible parent, is derived from a cross between the M2 and FT-1 lines. The cultivar IAC-100 is derived from the cross between IAC 78-2318 and IAC-12 (Rossetto *et al.*, 1995), the IAC 78-2318 line being a source of multiple-resistance genes against soybean-attacking insects (Lourenção *et al.*, 1987). The IAC-100 cultivar has been previously employed in several research studies as the standard genotype for insect resistance, both in Brazil (Pinheiro *et al.*, 2005) and abroad (McPherson *et al.*, 2007 and McPherson and Buss, 2007). The bi-parental cross and respective backcrosses were obtained from these cultivars. Subsequently, the following segregating generations were obtained: a) generation F_2_ and F_4_ from bi-parental combination; b) 30 F_2:3_ generation progenies from the bi-parental cross; c) 30 progenies from the second generation of respective inbred backcrosses, denominated BC_1_F_2:3_ and BC_2_F_2:3_. The cultivars BRS Celeste and MGBR-46 (Conquista) were used alongside the afore mentioned crosses. The experimental plot was installed on December 19^th^, 2002, in an experimental field at the Escola de Agronomia e Engenharia de Alimentos da Universidade Federal de Goiás (16° 36'S latitude, 49° 17'W longitude and 730 m above sea-level), in Goiânia, Goiás. The experimental field was open to natural stink bug infestation through the absence of insect chemical control. In order to increase natural crop infestation by insect migration at the final maturation or initial harvesting stages, crops were sown lately. Evaluation of stink bug infestation was carried out between the R_3_ and R_8_ stages (Fehr and Caviness, 1977), by the beating-tissue method with random sampling of the experimental area at ten day intervals (Gazzoni, 1998).

In order to evaluate the highest possible number of progenies, three experiments were installed using the random complete-block design, this consisting of 36 treatments (thirty regular and six common) with three replications. Thirty F_2:3_ and thirty progenies from each backcross (BC_1_F_2:3_ and BC_2_F_2:3_) were evaluated, these constituting the regular treatments. Common treatments were represented by sampling from F_2_ and F_4_ generations, the parent lines and the BRS Celeste and MGBR-46 (Conquista) cultivars. The plot consisted of one-meter lines, 0.5 m apart, with twelve plants apiece. Evaluation of the traits was undertaken in five plants per plot. The evaluated traits were: a) Grain filling period (GFP) - obtained by the difference in days between reproductive stages R_7_ and R_5_ (Fehr and Caviness, 1977) in the crop (Pinheiro *et al.*, 2005); b) Leaf retention (LR) - evaluated in the field by means of a scale ranging from 1 to 5, where 1 is equal to normal senescence and 5, stems and green leaves (unfeasible harvest) (Godoi *et al.*, 2002); c) Percentage index of pod damage (PIPD) - obtained from quantification of plant pods, as to quality, in good, intermediate or flat conditions, followed by transformation using the formula PIPD = (% intermediate pods) + % flat pods (Rossetto *et al.*, 1986); d) Percentage of spotted seeds (PSS) - visual evaluation where values ranging from 0 to 100% are attributed to the seeds according to damage caused by insects or colonization by yeast (*Nematospora coryli* Peglion). Statistical analysis of the data for the groups of experiments consisted basically of the individual analysis of variance for each experiment in random blocks, followed by grouped analysis of all experiments (Pimentel Gomes, 1990; Cruz and Carneiro, 2003).

### Genetic analysis of the means components

Estimates of mean components were carried out by the joint-scale method proposed by Cavalli (1952), which uses the weighted minimum squares method, whereby weighing factors are the inverted ratio of the variance of the means for each population evaluated. The variance of the means from the generations was obtained by dividing the treatment error mean square of the variance analysis grouped by their respective number of replications in each generation. The weighted analysis was used due to the fact that the estimates of the means are obtained with distinct precision among the different populations or families investigated (Mather and Jinks, 1984).

Genetic models were adjusted to means of the parent lines IAC-100 and FT-Estrela and their segregating generations F_2_ and F_4_ (as bulk), and F_3_ and the respective backcrosses in the second generation of inbreeding (BC_1_F_2:3_ and BC_2_F_2:3_), for those traits under investigation. Initially, it was predicted to use a simple genetic model of the dominant-additive type, involving the components m, [a] and [d], where, m is the average value between parents, [a] represents the algebraic sum of the additive effects of all distinct loci between the parents, and [d] represents the algebraic sum of dominance effects of all distinct loci between the parents. If the proposed model proves to be unsatisfactory for explaining genetic mechanisms controlling the traits being investigated, an alternative model may be used, this including non-allelic interaction parameters between pairs of loci, with the addition of components [aa], [ad] and [dd]. The additional components represent the epistatic interaction between homozygous loci from the additive x additive type, the epistatic interaction of the additive x dominant type and the epistatic interaction between heterozygous loci or of the dominant x dominant type, respectively. The proportion among the components m, [a], [d], [aa], [ad] and [dd], present in the evaluated generations, is shown in detail in Table S1.

Application of the joint-scale method is as described by Mather and Jinks (1984). Biometric analyses were performed using PROC IML proceeding from SAS (Sas Institute, 1998) software.

### Genetic analysis of the variance components

The study of the variances was performed admitting the absence of non-allelic and gene-linkage interactions. Therefore, it was considered that the total genetic variance ( 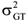) consists of additive ( 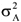) and dominant ( 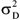) genetic components, where the ratio between them in successive inbred generations from a bi-parental cross follows a precise model dependent on the degree of endogamy (F). Thus, those components associated to 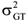 were obtained by the expression 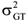 = (1 + F) 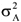 + (1 - F) 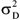. Total genetic variance consists of genetic variance among ( 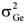) and within ( 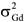) progenies. Genetic variance among progenies ( 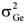) is obtained by 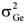 = (2F_n_) 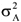 + F_n_(1-F_n_) 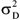, whereas this variance between progenies ( 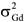) is calculated by 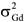 = (1 - F_n_) 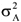 + (1 - F_n_) 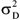, where F_n_ is the endogamy coefficient for generation n (Vencovsky and Barriga, 1992). For backcrosses, expected variances between progenies from the second successive inbreeding used in this work correspond to:


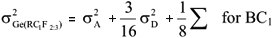


and


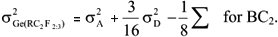


The expected variances within progenies are equivalent between BC_1_ and BC_2_, calculated by the expression:


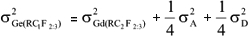


Those components associated to investigated generations are summarized in Table S2.

Thus, an approximation of genetic variance to studied traits is represented by the sum of the components of the additive variance ( 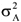) and variance due to dominant effects ( 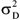), albeit, with the absence of epistatic interaction effects. Therefore, in the reference population F_2_, genetic variance contains 1/2 Σ(d^2^) + 1/4 Σ(h^2^) which is equivalent to 1/2 D + 1/4 H. As an alternative and according to the notation proposed by Vencovsky and Barriga (1992) it is shown that:


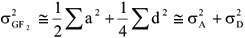


The use of the joint scale method has allowed us to obtain estimates of the parameters for the genetic model associated to observed variances, also allowing the verification of the adjustment of the additive-dominant genetic model to observed variances. The routine used to calculate model estimates by the iterative process is detailed in Mather and Jinks (1984) and Toledo (1991). The vector of phenotypic variances for the generations was represented by the mean squares of joint analysis of variance, where the diagonal matrix N is represented by the respective degrees of freedom associated to the mean squares. This procedure was carried out with MAPGEN (Ferreira, 2004) statistics software. Subsequent to calculation of the estimates of 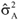, 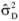, 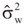 and 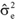, the heritability coefficients concerning reference F_2_ populations were calculated, in both broad and narrow senses, by using the following expressions:

Broad sense heritability (%):


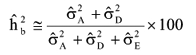


Narrow sense heritability (%):


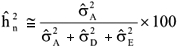


## Results and Discussion

### Genetic variability in the generations

A summary of individual analyses of variance for the investigated traits is presented in Tables S3 to S6. From this, it can be observed that the source of variation in treatment unfolds according to genetic group, thereby permitting recognition of the magnitude of variation in treatments within each group. This approach has allowed us to observe statistical differences among treatments for all investigated traits, thereby indicating the occurrence of genetic variability among genotypes. This result was expected, due to the presence of homozygous and segregating material in the treatments, and which showed a distinct response to the high level of stink bug infestation that occurred in the experimental area, crucially contributing to the differentiation and expression of genetic variability of the genotypes in their resistance- response.

**Figure 1:**
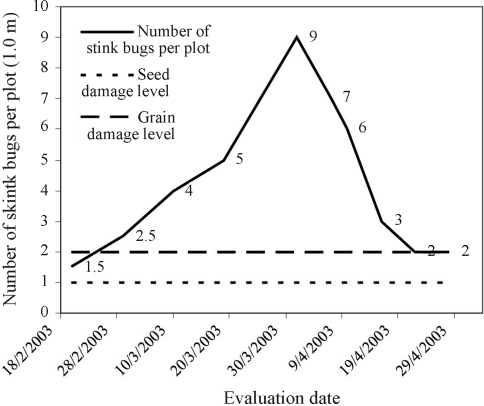
Fluctuation of the average stink bug population in the experiment during the period comprised between reproductive stages R_3_ and R_8_ of the evaluated genotypes.

Stink bug infestation in the experimental plot during the most susceptible period, between reproductive stages R_3_ and R_8_ (Fehr and Caviness, 1977), was always superior to the level normally accountable for economical losses in crops (Gazzoni, 1998), this reaching a maximum population equivalent to nine stink bugs per meter during stage R_6_ (Figure 1).

The results of joint-analysis of variance, with the respective values for mean squares associated to the source of variation, among and within treatment plots for resistance-associated traits GFP, LR, PIPD and PSS, are shown in Table 1. Furthermore, the partitioning of variation source treatments into genetic groups of interest was also carried out, along with the calculation of their related contrasts. Thus, the magnitude of observed phenotypic variability, among and within each group studied, was demonstrated.

The parents FT-Estrela (G1) and IAC-100 (G2) differed for all investigated traits (Table 1, contrast G1 *vs* G2), demonstrating genetic variability between parent lines in those traits pertaining to stink bug complex resistance. This contributes to generating genetic variability in segregating populations, due to gene recombination in inbred populations originating from crosses and backcrosses between genotypes. Concerning group decomposition, it was observed that there were statistically significant differences (p < 0.01) between F_2:3_ progenies for GFP and PIPD. For LR and PSS, these were statistically significant at 5%. There were no significant differences found in progenies regarding LR and PIPD traits in the BC_1_F_2:3_ group. The absence of statistical significance in the F test between treatments in this group is an indication of little genetic variability among the genotypes for these traits. However, as to the remaining traits, statistically significant differences were observed between progenies (Table 1). This behavior was expected, since greater genetic variability is foreseen between progenies F_2:3_ that are derived from a bi-parental cross where, in the F_2_ generation, all distinct loci between parental lines segregate. In contrast, the highest representation (75%) of a parental line in backcrosses induces lower levels of gene recombination in inbred generations. Consequently, the sampled progenies from backcrosses show a tendency towards presenting smaller differences one from the other.

It can be observed that the smaller mean values for the traits GFP, LR, PSS and PIPD, among the common treatments, occurred for the genotype IAC-100 (Table 2). The different behavior between IAC-100 and the susceptible material may be attributed to genetic resistance of its genotype (Rossetto *et al.*, 1995), which, when exposed to high stink bug infestation (Figure 1), presented less damage than that to susceptible genotypes. Similar results in IAC-100 behavior were reported by Souza and Toledo (1995), Godoi *et al.* (2002), Moura *et al.* (2003), Pinheiro *et al.* (2005) and McPherson *et al.* (2007).

### Genetic analysis of the mean components

The means and the degrees of freedom observed for the generations FT-Estrela (G1), IAC-100 (G2), F_2_, F_2:3_, BC_1_F_2:3_, BC_2_F_2:3_ and F_4_ are discriminated in Table 2. This information was used to obtain the joint scale test, verification of adjustment between genetic models and estimation of those genetic components associated to the means of the generations for the investigated traits.

In the group of experiments, statistically significant differences (p < 0,01) were observed in the mean contrasts between the parental lines FT-Estrela (G1) and IAC-100 (G2) for all those traits associated to stink bug resistance (Table 1), thereby indicating that the parental lines used for the crosses studied contrast for the investigated traits. This divergence between parent-lines is important in the context of the study, since, on applying the methodology of genetic analysis of mean components through the joint-scale test, the presupposition is that the parent-lines are completely homozygous lines displaying contrasting behavior for the trait (Mather and Jinks, 1984).

Genetic models adjusted to the generation means and their respective significance in the chi-square (χ^2^) test, the estimates of parameters, their significance and the errors associated to the estimates, are detailed in Table 3.

The additive-dominant genetic model (for testing parameters in relation to the mean value between parent lines (m), the sum of the additive effect of the genes [a] and the sum of the dominance effect among the alleles [d]) was adequate to explain the behavior of the means of those generations used in the grouped scale test, assuming the probability of 5% for the χ^2^ test for the traits GFP, LR, PIPD and PSS. Accumulated χ^2^ values were 3.8, 2.9, 6.1 and 10.1, respectively, on testing the additive-dominant model for these traits (Table 3).

Estimates of parameter [a], refering to the sum of gene additive effects, were positive and significant for the traits GFP, LR, PIPD and PSS, thus indicating that additive genetic effects condition the increase in traits. With the exception of the trait LR, results indicate the absence of dominant effects among resistance traits against the investigated stink bugs (Table 3).

It has been observed that the estimates of parameter [d], which represents the sum of deviations caused by dominance among alleles, were significant exclusively for LR. Similar results were found by Souza and Toledo (1995), who observed significant heterosis, although towards the resistant parent line, in contrast to our observations. However, the occurrence of null values in mean parameters, such as those found for the value of [d] in GFP, PIPD and PSS parameters (Table 3), does not mean the absence of the effect value, since this situation may be associated to gene dispersion in the parent lines, thereby leading to null effects in individual genes (Mather and Jinks, 1984).

In general, the heterosis effect occurs as a disturbing factor in selection processes, due to the difficulties it gives rise to in selecting really superior individuals at initial endogamy generation. Later, the effects of dominance are spread by the advance of inbred generations, as normally seen in soybean breeding programs. According to Pessoni *et al.* (1997), when dominant gene effects are present ([d], [ad] or [dd]), selection in early generations may not be adequate, especially if this occurs towards the susceptible genotype, since it may lead to the elimination of resistant genotypes, or vice-versa. In this situation, the author recommends the selection of traits with dominance effects in generations with higher levels of endogamy (from generations F_4_ or F_5_ on).

Therefore, based on the genetic studies of the means from the afore-cited generations for GFP, PIPD and PSS traits, it is expected that genetic gains may be obtained by plant selection in early endogamy generations (F_3_ or F_4_), due exclusively to the importance of additive gene effects on the expression of these traits. In contrast, for the LR trait which shows significant dominance effects, selection must be carried out in later generations with higher endogamy levels.

### Genetic analysis of variance components and heritability

Detailed information used in the joint-scale test is described in Table 4. This information applied to calculate the estimates of genetic and environmental variances for the investigated traits.

Phenotypic generation variances, represented by the mean squares of the sources of variation from joint-analysis of variation, are represented in Table 2. The mean squares of error among treatments and the mean squares within the pure lines were used to estimate the environmental variation within treatments.

The joint scale test used in this study favors estimating those genetic model parameters associated to observed variances. Moreover, it permits checking the adjustment of the additive-dominant genetic model to these (Toledo, 1991). The number of iterations that occur, on the convergence of parameter estimates to the adopted genetic model, corresponded to 11, 15, 11, 9, 14 and 10 to GFP, LR, PIPD and PSS, respectively (Table 5). The observed values reinforce information given by Mather and Jinks (1984), who suggest the use of a minimal number of 10 iterations, or alternatively, to execute them until values converge.

From the data on Table 5, it can be verified that the additive-dominant genetic model is sufficient to explain all genetic variability found in GFP, PIPD, PSS and LR traits, significant at 5% probability (p < 0.05). As with the results obtained in genetic analysis of the means, it was found that additive genetic variances ( 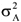) which represent the sum of the squares of the additive effects (α's) of those genes involved in trait expression, were more important for GFP and PIPD traits.

For the traits LR and PSS, dominance genetic variations ( 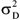) exhibited greater magnitudes than additive variance ( 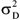) (Table 5). However, it has been observed that the estimates of the dominance genetic variance ( 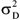) are associated to the errors of high estimates for all traits. Specifically, in the case of PIPD and on estimating the parameter, the result was negative. Based on the observed results, it may be suggested that the negative value for the 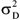 effect is null, mainly since, through analysis of the mean, the result indicates the absence of dominance effects for this trait. According to Pessoni *et al.* (1997), negative estimates may occur if the variance component shows low magnitude. Additionally, estimates may arise due to inadequate adjustment to the model, through being simultaneously associated to the sampling and evaluation processes used for this trait.

Estimated values for the heritability coefficient in the broad sense ( 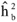) were 74.7%, 51.9%, 49.6% and 55.8%, and in the narrow sense ( 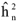) they were 36.1%, 19.9%, 49.6% and 20.3% for GFP, LR, PIPD and PSS traits, respectively (Table, 5). The suggestion is that the negative values were obtained for genetic variation when dominance equals zero, thus making it possible to obtain the heritability coefficient in both the broad and narrow sense for PIPD. Nevertheless, it should be noted that the 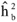 coefficient comprises all the genetic influences in its expression, instead of only the additive effects of additive genes. Thus, except for conditions where dominance effects are null, this cannot be used as a precise indicator for obtaining a precise estimation of selection gains. In other words, estimates of selection gains may be over-estimated by the use of this coefficient.

For the GFP, LR and PSS traits, the estimates of 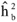 were superior to 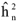. These estimates occurred due to the influence of the dominance component of the variance ( 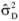) in the expression of the total phenotypic variance, which exhibited superior magnitudes in comparison to the effects of additive genetic variance in these traits. It is generally verified that an increase in magnitude in 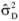 implies a decrease in 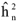 in the reference generation F_2_. Thus, it is observed that the selection of genotypes from initial generations for GFP, LR and PSS traits may be difficult due to the higher influence of dominance effects. According to Vencovsky and Barriga (1992), selection for low heritability traits, or for those with dominance, is ineffective when carried out in early generations. For this reason, selection based on these traits is more effective when undertaken in subsequent generations. In this way, the occurrence of heterozygotes is reduced and, consequently, dominance variation is maximized, while the available additive variance for selection is increased, thereby providing higher possibilities of selection gains for the trait.

According to Brogin *et al.* (2003), heritability values are considered small when inferior to 30%, intermediate when between 30% and 60% and high when superior to 60%. The 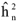 values obtained for GFP and PIPD were over 30%, and therefore can be considered to be intermediate to high. According to Reis *et al.* (2002), heritability values in this magnitude range may be associated to lower complexity in genetic control of the trait, and probably the additive effects represent a higher proportion in total phenotypic variation, with few genes involved in its expression. These results may be associated to high heritability values, possibly due to high genetic variability among the evaluated genotypes and efficient environmental control achieved in the experimental field, this being reflected in CV (Table 1), and on considering that heritability is a genetic factor that is specific for a given population, trait and field conditions from which it is obtained.

According to Brogin *et al.* (2003), traits with heritability estimates higher than 30% allow for genetic gains through selection in initial generations of endogamy, such as generations F_3_ or F_4_. In the present study, the estimate of 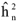 was 36% for the GFP trait, although dominance gene effects are of a higher magnitude than additive ones. Thus, under these conditions, the selection of resistant genotypes based on the GFP trait should be carried out in advanced endogamy generations.

Therefore, for GFP, LR and PSS traits, which exhibit 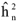 values of 36.06%, 19.94% and 20.27%, respectively, in the reference F_2_ generation, besides significant dominance gene effects, it is recommended to select genotypes in populations with higher endogamy levels. In this way, it is possible to increase the magnitude of available additive variance and decrease gene dominance effects on the trait itself. According to Silva *et al.* (2004), in theory, it is considered that an F_5_ generation individual presents enough homozygosis levels to allow for selection, mainly due to the absence of significant additions to the level of homozygous individuals in future generations which would imply longer periods for selection.

The obtained 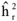 value for PIPD was 49.61%. This means that it is possible to obtain genetic gains from selection in initial generations of endogamy, such as in generations F_3_ or F_4_. However, there are significant difficulties in the evaluation of this trait, due to the need for a representative sample of pods per plant. This can be problematic, since in these stages there are generally many genotypes to be evaluated.

Based on the observed results from genetic analyses of means and variances, as well as estimates of heritability coefficients, it can be concluded that the best strategy for obtaining stink bug resistant genotypes is selection of the PIPD trait in early generations (F_3_ or F_4_), followed by selection for GFP, LR and PSS in following generations with higher endogamy levels.

## Supplementary Material

The following online material is available for this article:

Table S1 - Genetic components of the expected means for parent lines and their segregating generations, involving additive, dominance and di-genic epistatic interactions used in the joint scale test proposed by Cavalli (1952).
Table S2 - Genetic and environmental components associated to phenotypic variances of inbred lines and segregating generations, without considering the influence of epistatic effects on trait expression.
Table S3 - Summary of analysis of variance, with those mean squares associated to variation among the means and within plots from their respective sources of variation, for the trait grain filling period (days).
Table S4 - Summary of analysis of variance, with those mean squares associated to variation among the means and within plots from their respective sources of variation for the trait leaf retention (grade).
Table S5 - Summary of analysis of variance, with those mean squares associated to variation among the means and within the plots from the respective sources of variation for the trait percentage index of pod damage in soybeans (%).
Table S6 - Summary of analysis of variance, with those mean squares associated to variation among the means and within the plots from their respective sources of variation for the trait percentage of spotted soybean seeds (%).
This material is available as part of the online article from http://www.scielo.br/gmb.

## Figures and Tables

**Table 1:** Summary of the joint analysis of variance, with the mean squares associated to variation among the means and within the plots, from their respective sources of variation, for traits associated to resistance against the stink bug complex (GFP, LR, PIPD and PSS)^1^ in soybean.

SV	GFP (days)			LR (grade)^2^			PIPD (%)^3^			PSS (%)^3^	
	DF	MSQ		DF	MSQ		DF	MSQ		DF	MSQ
Blocks/experiment	6	20.8561**		6	0.2711**		6	0.0197**		6	0.0875^ns^
Experiments (E)	2	3.7267^ns^		2	0.0028^ns^		2	0.0186**		2	0.0079^ns^
E x Common treat.	10	6.8378^ns^		10	0.0911^ns^		10	0.0094^ns^		10	0.0579^ns^
Treatments/E	95	21.5287**		95	0.1698**		95	0.0128**		95	0.1657**
F_2:3_	29	15.4427**		29	0.1136*		29	0.0116**		29	0.0906*
BC_1_F_2:3_ (BC_1_)	29	10.8686**		29	0.1744**		29	0.0130**		29	0.0581^ns^
BC_2_F_2:3_ (BC_2_)	29	16.0359**		29	0.0846ns		29	0.0071^ns^		29	0.1292**
Common treat.	5	81.9804**		5	0.6643**		5	0.0392**		5	0.5799**
Groups	3	127.7741**		3	0.5291**		3	0.0316**		3	1.4923**
G_1_*vs.* G_2_^4^	(1)	76.0556**		(1)	0.8823**		(1)	0.1352**		(1)	1.4857**
BC_1_*vs.* BC_2_	(1)	285.3848**		(1)	0.6341**		(1)	0.0658**		(1)	2.1842**
Error among	209	5.0292		209	0.0686		207	0.0054		207	0.0510
Error within	1284	10.463		1286	0.1129		1249	0.0109		1246	0.0852
Within F_2:3_	(360)	12.534		(360)	0.1154		348	0.0104		348	0.0967
Within BC_1_	(357)	9.297		(357)	0.1232		351	0.0106		346	0.0831
Within BC_2_	(354)	10.326		(356)	0.1137		340	0.0105		340	0.0812
Within F_2_	(36)	13.944		(36)	0.1402		34	0.0164		36	0.1105
Within F_4_	(36)	20.622		(36)	0.1162		35	0.0161		36	0.0996
Within IL	141	4.99		141	0.0704		141	0.0110		140	0.0609

Means	29.23			1.73			0.53			0.89	
CV(%)	7.67			15.15			13.96			25.29	

* and **: significant at 5% and 1% of probability by the F Test, respectively; ^1^GFP (grain filling period), RF (leaf retention), PIPD (percentage index of pod damage) and PSS (percentage of spotted seeds); ^2^Raw data were transformed by $\sqrt{x+0.5}$; ^3^Raw data were transformed by $\arcsin\sqrt{x/100}$; ^4^G_1_: FT-Estrela and G_2_: IAC-100.

**Table 2:** Joint-scale test information (Mather and Jinks, 1984) involving the parent lines FT-Estrela (G1) and IAC-100 (G2) and their segregating generations F_2_, progenies F_2:3_, BC_1_F_2:3_, BC_2_F_2:3_ and F_4_, for traits associated to resistance against the stink bug complex (GFP, LR, PIPD and PSS)^1^ in soybean.

Generations	GFP (days)			LR (grade^2^)			PIPD (%)^3^			PSS (%)^3^	
	N^4^	Mean		N	Mean		N	Mean		N	Mean
FT-Estrela	9	30.689		9	1.930		9	0.605		9	1.203
IAC-100	9	26.578		9	1.488		9	0.431		9	0.629
F_2_	9	29.622		9	1.795		9	0.522		9	0.997
F_2:3_	90	28.724		90	1.716		90	0.517		90	0.909
BC_1_F_2:3_	90	30.407		90	1.758		89	0.545		89	0.9437
BC_2_F_2:3_	89	27.846		89	1.638		88	0.504		88	0.719
F_4_	9	29.089		9	1.632		9	0.541		9	0.991

^1^GFP (grain filling period); LR (leaf retention); PIPD (percentage index of pod damage); PSS (percentage of spotted seeds). ^2^Raw data transformed by $\sqrt{x+0.5}$. ^3^Raw data transformed by $\arcsin\sqrt{x/100}$. ^4^Number of sampled plots.

**Table 3:** Genetic models adjusted to the means of the FT-Estrela (G1) and IAC-100 (G2) parent lines and their segregating populations F_2_, F_3:2_, BC_1_F_3:2_, BC_2_F_3:2_ and F_4_.

Model^1^	Traits^2^			
	GFP (days)	LR^3^ (scale)	PSS^4^ (%)	PIPD^4^ (%)
m	28.998** ± 0.2618	1.6799** ± 0.0007	0.8159** ± 0.0431	0.5257** ± 0.0109
[a]	2.417** ± 0.2748	0.1490** ± 0.0008	0.2436** ± 0.0454	0.0543** ± 0.0115
[d]	-0.035^ns^ ± 1.3906	0.1716** ± 0.0200	0.3200^ns^ ± 0.2289	-0.0207^ns^ ± 0.0580
χ^2^	3.77^ns^	2.91^ns^	10.06^ns^	6.09^ns^
DF	4	4	4	4

* and **: significant at 5% and 1% of probability by the *t* Test, respectively. ^1^m = mean of homozygous lines derived from F_2_; [a] = estimate of the additive gene effect; [d] = estimate of gene dominance deviation. ^2^GFP (grain filling period); LR (leaf retention); PIPD (percentage index of pod damage); PSS (percentage of spotted seeds). ^3^Raw data transformed by $\sqrt{x+0.5}$. ^4^Raw data transformed by $\arcsin\sqrt{x/100}$.

**Table 4:** Information employed in the joint-scale test for obtaining estimates of genetic additive ( ${\hat{\sigma }}_{\text{A}}^{2}$), dominance ( ${\hat{\sigma }}_{\text{D}}^{2}$) and environmental ( ${\hat{\sigma }}_{\text{E}}^{2}$) variances, using weighted least-squares (Mather and Jinks, 1984) for traits associated to resistance against the stink bug complex (GFP, LR, PIPD and PSS)^1^ in soybean.

SV^2^	GFP			LR^3^			PIPD^4^			PSS^4^	
	DF	MSQ^5^		DF	MSQ^5^		DF	MSQ^5^		DF	MSQ^5^
$\sigma_{{\text{FT(F}}_{\text{2}}\text{)}}^{2}$	36	13.9440		36	0.1402		34	0.0164		36	0.1105
$\sigma_{{\text{Fe(F}}_{\text{2:3}}\text{)}}^{2}$	29	15.4427		29	0.1136		29	0.0116		29	0.0906
$\sigma_{{\text{Fd(F}}_{\text{2:3}}\text{)}}^{2}$	360	12.5340		360	0.1154		348	0.0104		348	0.0967
$\sigma_{{\text{Fe(RC}}_{1}{\text{F}}_{\text{2:3}}\text{)}}^{2}$	29	10.8686		29	0.1744		29	0.0130		29	0.0581
$\sigma_{{\text{Fd(RC}}_{1}{\text{F}}_{\text{2:3}}\text{)}}^{2}$	357	9.2970		357	0.1232		351	0.0106		346	0.0831
$\sigma_{{\text{Fe(RC}}_{2}{\text{F}}_{\text{2:3}}\text{)}}^{2}$	29	16.0359		29	0.0846		29	0.0071		29	0.1292
$\sigma_{{\text{Fd(RC}}_{2}{\text{F}}_{\text{2:3}}\text{)}}^{2}$	354	10.3260		356	0.1137		340	0.0105		340	0.0812
$\sigma_{{\text{FT(F}}_{\text{4}}\text{)}}^{2}$	36	20.6220		36	0.1162		35	0.0161		36	0.0996
MSQ error among means	209	5.0290		209	0.0686		207	0.0054		207	0.0510
MSQ error within plot	141	4.9900		141	0.0704		141	0.0110		140	0.0609

^1^GFP (grain filling period) - LR (leaf retention) - PIPD (percentage index of pod damage) - PSS (percentage of spotted seeds). ^2^Sources of variation from joint-analysis of variance and its respective degrees of freedom and mean squares. ^3^Raw data transformed to $\sqrt{x+0.5}$. ^4^Raw data transformed to $\arcsin\sqrt{x/100}$. ^5^Mean squares of sources of variation from variance joint-analysis of variance (Table 1).

**Table 5:** Estimates of additive ( ${\hat{\sigma }}_{\text{A}}^{2}$), dominance ( ${\hat{\sigma }}_{\text{D}}^{2}$) and environmental ( ${\hat{\sigma }}_{\text{E}}^{2}$) genetic variances, obtained by weighted least squares. Estimates of heritability coefficients in the broad ( ${\hat{h}}_{b}^{2}$) and narrow ( ${\hat{h}}_{b}^{2}$) senses, and adherence test of the additive-dominant model applied to phenotypic variances of the evaluated groups.

Parameters	Traits^1^			
	GFP (days)	LR (grade)^2^	PIPD (%)^3^	PSS (%)^3^
${\hat{\sigma }}_{\text{A}}^{2}$	7.1780 ± 2.507	0.0295 ± 0.033	0.0054 ± 0.002	0.0240 ± 0.017
${\hat{\sigma }}_{\text{D}}^{2}$	7.6822 ± 3.816	0.0474 ± 0.063	-0.0033 ± 0.003	0.0420 ± 0.032
${\hat{\sigma }}_{\text{E}}^{2}$	5.0448	0.0711	0.0055	0.0523
${\hat{h}}_{b}^{2}$	74.66	51.94	49.61	55.80
${\hat{h}}_{n}^{2}$	36.06	19.94	49.61	20.27
Iterations	11	15	11	9
χ^2^	6.43	17.07	6.75	7.57
DF	5	5	5	5
Probability	0.26	0.04	0.24	0.18

^1^GFP (grain filling period) - LR (leaf retention) - PIPD (percentage index of pod damage) - PSS (percentage of spotted seeds). ^2^Raw data transformed by $\sqrt{x+0.5}$. ^3^Raw data transformed by $\arcsin\sqrt{x/100}$.
