# Supplementary material for: Genetic parameters and selection strategies for soybean genotypes resistant to the stink bug-complex
Source: Genet Mol Biol. 2009 Jun 1;32(2):328–36. doi: 10.1590/S1415-47572009000200020 (PMC3036932; doi:10.1590/S1415-47572009000200020)
Supplement: Supplementary file 1 — Table S1 - Genetic components of the expected means for parent lines and their segregating generations, involving additive, dominance and di-genic epistatic interactions used in the joint scale test proposed by Cavalli (1952). Table S2 - Genetic and environmental components associated to phenotypic variances of inbred lines and segregating generations, without considering the influence of epistatic effects on trait expression. Table S3 - Summary of analysis of variance, with those mean squares associated to variation among the means and within plots from their respective sources of variation, for the trait grain filling period (days). Table S4 - Summary of analysis of variance, with those mean squares associated to variation among the means and within plots from their respective sources of variation for the trait leaf retention (grade). Table S5 - Summary of analysis of variance, with those mean squares associated to variation among the means and within the plots from the respective sources of variation for the trait percentage index of pod damage in soybeans (%). Table S6 - Summary of analysis of variance, with those mean squares associated to variation among the means and within the plots from their respective sources of variation for the trait percentage of spotted soybean seeds (%). This material is available as part of the online article from http://www.scielo.br/gmb. [file gmb-32-2-328-suppl1.pdf]

Table S1 – Genetic components of the expected means for parent lines and their segregating generations, involving additive, dominance and di-genic epistatic interactions used in the joint scale test proposed by Cavalli (1952).

Geration	Genetic components [1]					
	m	[a]	[d]	[aa]	[ad]	[dd]
FT-Estrela (Parent line 1)	1	1	0	1	0	0
IAC-100 (Parent line 2)	1	-1	0	1	0	0
F_2	1	0	½	0	0	¼
F_3	1	0	¼	0	0	$\frac{1}{16}$
$BC_1F_{2:3}$	1	½	$\frac{1}{8}$	¼	$\frac{1}{16}$	$\frac{1}{64}$
$BC_2F_{2:3}$	1	- ½	$\frac{1}{8}$	¼	$-\frac{1}{16}$	$\frac{1}{64}$
F_4	1	0	$\frac{1}{8}$	0	0	$\frac{1}{64}$

[1] m = mean of F_2-derived homozygous lines; [a] = estimate of additive gene effect; [d] = estimate of dominance deviation, [aa] = estimation of di-gene epistatic interactions of the additive x additive type; [ad] = estimate of the di-gene epistatic interactions of the additive x dominant type; [dd] = estimate of the di-genic epistatic interactions of the type dominant x dominant.

Table S2 – Genetic and environmental components associated to phenotypic variances of inbred lines and segregating generations, without considering the influence of epistatic effects on trait expression.

Generations	$\hat{\sigma}^2_A$	$\hat{\sigma}^2_D$	$\sum ad$	$\hat{\sigma}^2_w$	$\hat{\sigma}^2_e$
$\sigma^2_{FT(F_2)}$	1	1	0	1	0
$\sigma^2_{Fe(F_{2:3})}$	1	$1/4$	0	$1/\bar{n}$	1
$\sigma^2_{Fd(F_{2:3})}$	$1/2$	$1/2$	0	1	0
$\sigma^2_{Fe(RC_1F_{2:3})}$	1	$3/16$	$1/8$	$1/\bar{n}$	1
$\sigma^2_{Fd(RC_1F_{2:3})}$	$1/4$	$1/4$	0	1	0
$\sigma^2_{Fe(RC_2F_{2:3})}$	1	$3/16$	$-1/8$	$1/\bar{n}$	1
$\sigma^2_{Fd(RC_2F_{2:3})}$	$1/4$	$1/4$	0	1	0
$\sigma^2_{FT(F_4)}$	$7/4$	$7/16$	0	1	0
MSQ Error between the plots	0	0	0	$1/\bar{n}$	1
MSQ Error within the plot	0	0	0	1	0

$\bar{n}$: harmonic mean of the number of plants per plot; $\hat{\sigma}^2_E = \hat{\sigma}^2_w/\bar{n} + \hat{\sigma}^2_e$.

Table S3 – Summary of analysis of variance, with those mean squares associated to variation among the means and within plots from their respective sources of variation, for the trait grain filling period (days).

SV	Experiment 1		Experiment 2		Experiment 3	
	DF	MSQ	DF	MSQ	DF	MSQ
Blocks	6	3.9932ns	6	36.3392**	6	22.2359**
Treatments	35	22.9248**	35	19.0386**	35	18.5253**
$F_{2:3}$	9	8.5287ns	9	22.9495**	9	15.3636**
$BC_1F_{2:3}$ (BC_1)	9	11.0317*	9	11.6043ns	9	12.3147**
$BC_2F_{2:3}$ (BC_2)	9	15.4168**	9	18.1636**	9	16.595**
Bulks	1	2.6667ns	1	5.2267ns	1	3.8400ns
Inbred lines (IL)	3	66.4033**	3	24.0756*	3	49.4167**
Groups	4	67.8136**	4	28.6101**	4	23.7875**
G_1 *vs* G_2 [1]	(1)	11,2067ns	(1)	20.1667ns	(1)	52.8067**
BC_1 *vs* BC_2	(1)	159.3100**	(1)	66.1500**	(1)	71.7227**
Error among means	69	4.42	70	6.2718	70	4.3872
Error within plots	423	9.8531	429	12.9249	432	10.0657
Within $F_{2:3}$	120	13.2400	120	15.7567	120	12.2233
Within $BC_1F_{2:3}$	117	9.3641	120	9.9167	120	8.6133
Within $BC_2F_{2:3}$	114	7.6550	120	15.2900	120	9.5000
Within F_2	12	8.6667	12	12.9667	12	20.2000
Within F_4	12	15.7667	12	22.1667	12	23.9333
Within IL	48	6.6167	45	4.6133	48	3.7167
Means	28.45		29.21		30.02	
CV (%)	7.39		8.57		6.98	

* and **: significant at 5% and 1% of probability by F Test, respectively.

[1] G_1: FT-Estrela and G_2: IAC-100.

Table S4 – Summary of analysis of variance, with those mean squares associated to variation among the means and within plots from their respective sources of variation for the trait leaf retention (grade).

SV	Experiment 1		Experiment 2		Experiment 3	
	DF	MSQ	DF	MSQ	DF	MSQ
Blocks	2	0.0486ns	2	0.539**	2	0.2257ns
Treatments	35	0.2244**	35	0.4397**	35	0.1227*
$F_{2:3}$	9	0.0987ns	9	0.0897ns	9	0.1708*
$BC_1F_{2:3}$ (BC_1)	9	0.323**	9	0.0635ns	9	0.167*
$BC_2F_{2:3}$ (BC_2)	9	0.0728ns	9	0.1285*	9	0.047ns
Bulks	1	0.1652ns	1	0.0467ns	1	0.0007ns
Inbred line (IL)	3	0.6171**	3	0.1971*	3	0.2154*
Groups	4	0.3347**	4	0.4288**	4	0.0463ns
G_1 *vs* G_2 [2]	(1)	0.9292**	(1)	0.1572ns	(1)	0.071ns
BC_1 *vs* BC_2	(1)	0.8301**	(1)	0.3276*	(1)	0.1221ns
Error among the means	69	0.0734	70	0.0558	70	0.07651
Error within the plot	425	0.0973	429	0.1173	432	0.1238
Within $F_{2:3}$	120	0.0877	120	0.1202	120	0.1383
Within $BC_1F_{2:3}$	117	0.1086	120	0.1429	120	0.1178
Within $BC_2F_{2:3}$	116	0.1134	120	0.1041	120	0.1235
Within F_2	12	0.0650	12	0.1748	12	0.1808
Within F_4	12	0.1779	12	0.0523	12	0.1183
Within IL	48	0.0424	45	0.0788	48	0.0905
Means	1.71		1.72		1.75	
CV (%)	15.84		13.74		15.77	

[* and **]: significant at 5% and 1% of probability by F Test, respectively.

[1] Raw data transformed by $\sqrt{(x+0.5)}$.

[2] G_1: FT-Estrela and G_2: IAC-100.

Table S5 – Summary of analysis of variance, with those mean squares associated to variation among the means and within the plots from the respective sources of variation for the trait percentage index of pod damage in soybeans (%).

SV	Experiment 1		Experiment 2		Experiment 3	
	DF	MSQ	DF	MSQ	DF	MSQ
Blocks	2	0.0161ns	2	0.0272**	2	0.0157*
Treatments	35	0.0111*	35	0.0121**	35	0.0141**
$F_{2:3}$	9	0.0093ns	9	0.0076ns	9	0.0165**
$BC_1F_{2:3}$ (BC_1)	9	0.0093ns	9	0.0131*	9	0.0168**
$BC_2F_{2:3}$ (BC_2)	9	0.0072ns	9	0.0072ns	9	0.0090ns
Bulks	1	0.0021ns		0.0006ns	1	0.0084ns
Inbred lines (IL)	3	0.0418**	3	0.0218**	3	0.0116ns
Groups	4	0.0133ns	4	0.0245**	4	0.0179*
G_1 *vs* G_2 [2]	(1)	0.1180**	(1)	0.0231*	(1)	0.0200*
BC_1 *vs* BC_2	(1)	0.0525**	(1)	0.0524**	(1)	0.0002ns
Error among the means	69	0.0060	69	0.0052	69	0.0050
Error within the plots	413	0.0099	411	0.0115	425	0.0112
Within $F_{2:3}$	115	0.0113	114	0.0097	119	0.0102
Within $BC_1F_{2:3}$	117	0.0115	114	0.0131	120	0.0074
Within $BC_2F_{2:3}$	110	0.0082	114	0.0106	116	0.0127
Within F_2	12	0.0140	12	0.0116	10	0.0249
Within F_4	11	0.0066	12	0.0072	12	0.0337
Within IL	48	0.0068	45	0.0155	48	0.0111
Means	0.52		0.53		0.53	
CV (%)	14.87		13.79		13.21	

* and **: significant at 5% and 1% of probability by F Test, respectively.

[1] Raw data transformed by $\text{arcsen}\sqrt{(x/100)}$

[2] G_1: FT-Estrela and G_2: IAC-100.

Table S6 - Summary of analysis of variance, with those mean squares associated to variation among the means and within the plots from their respective sources of variation for the trait percentage of spotted soybean seeds (%).

SV	Experiment 1		Experiment 2		Experiment 3	
	DF	MSQ	DF	MSQ	DF	MSQ
Blocks	2	0.0349ns	2	0.0902ns	2	0.1375ns
Treatments	35	0.2170**	35	0.1466**	35	0.1027*
$F_{2:3}$	9	0.1197ns	9	0.0635ns	9	0.0961ns
$BC_1F_{2:3}$ (BC_1)	9	0.0596ns	9	0.0425ns	9	0.7589ns
$BC_2F_{2:3}$ (BC_2)	9	0.1283*	9	0.1936**	9	0.0771ns
Bulks	1	0.0028ns		0.0154ns	1	0.0087ns
Inbred lines (IL)	3	0.6841**	3	0.2818**	3	0.1084ns
Groups	4	0.6558**	4	0.3928**	4	0.2391**
G_1 *vs* G_2 [2]	(1)	1.3379**	(1)	0.3687**	(1)	0.1206ns
BC_1 *vs* BC_2	(1)	1.5601**	(1)	0.5499**	(1)	0.3240*
Error among means	69	0.0604	69	0.0341	69	0.0585
Error within the plots	412	0.0896	411	0.0865	423	0.0795
Within $F_{2:3}$	114	0.0951	116	0.1067	118	0.0886
Within $BC_1F_{2:3}$	115	0.1005	113	0.0857	118	0.0636
Within $BC_2F_{2:3}$	111	0.0846	114	0.0791	115	0.0800
Within F_2	12	0.0532	12	0.1350	12	0.1431
Within F_4	12	0.1646	12	0.0655	12	0.0689
Within IL	48	0.0527	44	0.0469	48	0.0820
Means	0.85		0.95		0.88	
CV (%)	28.96		19.49		27.40	

[* and **]: significant at 5% and 1% of probability by F Test, respectively.

[1] Raw data transformed by $\text{arcsen}\sqrt{(x/100)}$

[2] G_1: FT-Estrela and G_2: IAC-100.
